# Identification of a novel *TBX5* mutation in a Chinese family with rare symptoms of Holt–Oram syndrome

**DOI:** 10.1016/j.heliyon.2022.e11774

**Published:** 2022-11-21

**Authors:** Xia Li, Weizhe Shi, Xuejiao Ding, Jingchun Li, Yiqiang Li, Jianping Wu, Zhe Yuan, Tianying Nong, Hongwen Xu, Mingwei Zhu

**Affiliations:** a Guangzhou Institute of Pediatrics, Guangzhou Women and Children’s Medical Center, Guangzhou Medical University, Guangzhou, China; b Department of Pediatric Orthopedics, Guangzhou Women and Children’s Medical Center, Guangzhou Medical University, Guangzhou, China

**Keywords:** Holt–Oram syndrome, Common atrium, Whole exome sequencing, TBX5, Novel mutation

## Abstract

Holt–Oram syndrome (HOS) is a rare autosomal dominant disorder characterized by skeletal abnormalities of the upper limbs and often cardiac malformations. We investigated a Chinese family with clinical features suggestive of HOS. Clinical examinations revealed that both the proband and his father had anomalies in the upper limbs and heart. The proband had a rare common atrium. Whole exome sequencing detected a novel small–insertion mutation (c.680_681insCTGAGAATAAT; p.Ile227fs∗) in *TBX5* gene, the known disease gene for HOS. The mutation cosegregated with HOS phenotypes in the family and was predicted to cause frameshift, resulting in a truncated protein. In this study, we described a rare HOS case with common atrium. A novel small–insertion in *TBX5* coding sequence was identified and speculated to be the disease–causing genetic variant in the family. Our finding expands the clinical feature spectrum and genetic aetiology spectrum of HOS.

## Teaser

Holt–Oram syndrome; Common atrium; Ehole exome sequencing; TBX5; Novel mutation.

## 1 Introduction

Holt–Oram Syndrome (HOS, OMIM 142900) is a rare autosomal dominant disorder characterized mainly by upper–limb defects and congenital heart malformation (Heinritz et al., 2005; Holt and Oram, 1996). Upper limb anomalies in HOS patients are completely penetrant and mainly manifest as defects in the preaxial radial ray, resulting predominantly in thumb abnormalities (Basson et al., 1994; Newbury-Ecob et al., 1996). Cardiac anomalies are present in 75% of HOS patients, with atrial septal defects and ventricular septal defects as the most common features. HOS symptoms severity varies, even within members of the same family (Lehner et al., 2003). To date, more than 120 mutations in the *TBX5* gene have been identified that cause more than 85% of HOS cases (Basson et al., 1997; Krauser et al., 2022; Li et al., 1997). However, whether there is a genotype–phenotype correlation in HOS remains unclear (Dreßen et al., 2016; Vanlerberghe et al., 2019; Yang et al., 2000).

We reported information about a Chinese family with two members suffering from HOS. The proband had a rare heart malformation phenotype—common atrium and severe upper limb deformities. His father presented with mild deformities in the heart and upper limbs. Through whole exome sequencing and genetic analysis, we identified a novel small–insertions mutation of *TBX5*, NM_181486.4: c.680_681insCTGAGAATAAT, which cosegregated with HOS phenotypes. This mutation leads to a frameshift and the introduction of a stop codon (X) immediately after residue Ile 227, resulting in a truncated protein. Our study described a new case with rare HOS phenotypes and identified a novel pathogenic mutation of *TBX5*.

## 2 Materials and methods

### 2.1 Patients and ethics

The proband, a 5-year-old boy, was referred to Guangzhou Women and Children’s Medical Center for the treatment of upper limb deformities. Clinical and radiographic examinations were performed. At the age of 2 years and 3 months, he was diagnosed with congenital heart disease characterized mainly by a common atrium. His father presented with mild deformities in the heart and upper limbs. The study protocols were approved by the Human Ethics Committee of the Guangzhou Women and Children’s Medical Center. Written informed consent was obtained from each participant or their legal custodians.

### 2.2 Whole-exome sequencing

Genomic DNA (gDNA) was extracted from the peripheral blood of all subjects with a Blood DNA Kit (Omega, USA) according to the manufacturer’s instructions. Whole exome sequencing (WES) was performed at the Novogene (Beijing, China) as previously described (Xian et al., 2021).

### 2.3 Filtering of genetic variants

All variants detected by WES were filtered following a pipeline: 1) exclusion of variants with a frequency greater than 1% in any of the four databases (1000g_all, esp6500siv2_all, gnomAd_ALL and gnomAD_EAS), 2) exclusion of variants that were not in the coding (exonic) region or splicing region (splicing site ± 10 bp), 3) exclusion of synonymous SNPs that were not predicted by dbscSNV to affect splicing, and 4) retention of variants that were predicted by at least two of four prediction tools (SIFT, Polyphen, MutationTaster, and CADD) to be deleterious and variants that are predicted to affect splicing.

### 2.4 Sanger sequencing

Conventional Sanger sequencing was performed to validate the pathogenic mutations. gDNA extracted from peripheral blood was used as a template. The following primers were used: 5′-TGGAATGATGCAGAGTATGAC-3′ (forwards) and 5′-ACCACATGTGAAGGTTATCAG-3′ (reverse). PCR was performed with High fidelity PrimeSTAR Max DNA Polymerase (Takara, Beijing, China). The qualified products (534 bp) were sent to Shanghai Sangon Biotech (Shanghai, China) for sequencing.

## 3 Results

**Figure 1:**
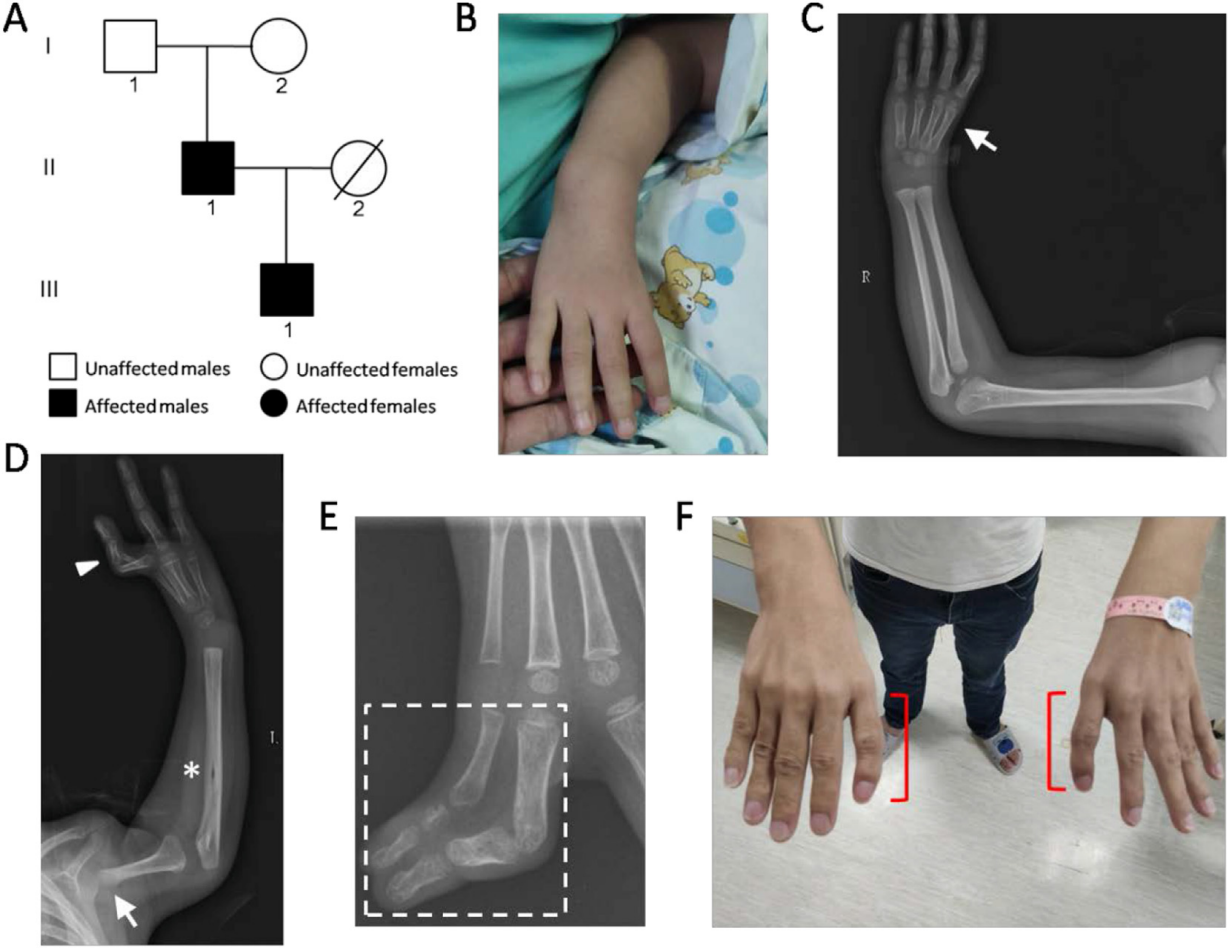
A Chinese family with Holt–Oram syndrome. (A) The pedigree. III:1 is the proband. (B) The thumb of the proband’s right hand is missing. (C) X-radiograph of the proband’s right upper limb. The arrow points to the missing thumb. (D) X-radiograph of the proband’s left upper limb, which indicates the absence of the proximal humerus (arrow) and the absence of the radius (asterisk), and 2/3 syndactyly (arrowhead). (E) X-radiograph of the proband’s left hand. The dotted line encompasses the region indicating 2/3 syndactyly. (F) Bilateral triphalangeal thumbs of the father of the proband.

The proband (III:1) was a 5-year-old boy (Figure 1A). Clinical and radiographic examinations showed bilateral upper limb defects. The right thumb was absent (Figure 1B, C). On the left, the deformity was more severe, characterized by dysplasia of the distal humerus, absence of the proximal humerus, dislocation of the shoulder, absence of the radius, limited extension of the elbow, absence of the phalanges of the thumb and 2/3 syndactyly (Figure 1D, E). In addition, at the age of 2 years and 3 months, the proband was diagnosed with congenital heart malformation, characterized by common atrium, unroofed coronary sinus syndrome, and persistent left superior vena cava. The systolic and diastolic functions of his left ventricle were normal. All of these features suggested HOS. The father (II:1) of the proband presented with mild HOS symptoms, mainly including bilateral triphalangeal thumbs and interventricular septal defects of the heart (Figure 1F). The mother (II:2) was deceased, but she did not have any signs of limb or heart abnormalities according to the father’s statement. The grandfather (I:1) and grandmother (II:2) also had no symptoms of HOS.

**Figure 2:**
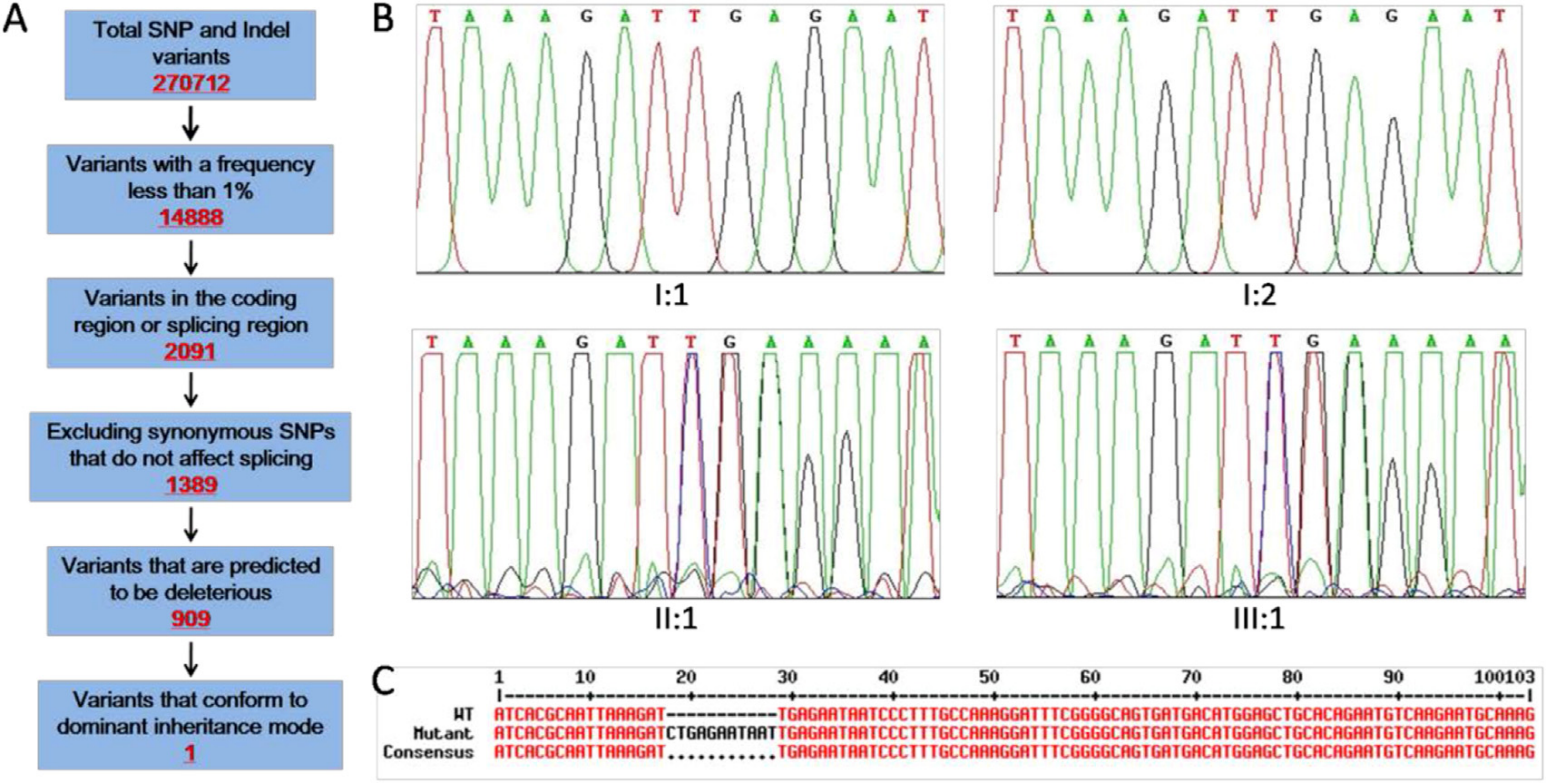
Identification of a novel mutation in the *Tbx5* gene cosegregated with HOS phenotypes. (A) Schematic representation of the filtering process of WES data. (B) Sanger sequencing results from blood genomic DNA. All affected individuals (II:1 and III:1) carry the heterozygous mutation. The unaffected I:1 and I:2 individuals were used as controls. (C) Alignment indicates that an 11 bp nucleotide, CTGAGAATAAT, was inserted into exon 7 in the mutant *Tbx5* gene.

To comprehensively identify the genetic lesion causing HOS in the family, we collected gDNA samples from all five family members and performed whole exome sequencing. A total of 270,712 genetic variants, including 233377 SNPs (**s**ingle **n**ucleotide **p**olymorphism) and 37,335 indels (small **in**sertion or **del**etion, <50 bp), were detected. After data filtering following the pipeline (Figure 2A), 762 SNPs and 147 indels were retained. Then, we screened for variants that conformed to dominant inheritance mode. Finally, a novel small–insertion mutation of *TBX5*, NM_181486.4: c.680_681insCTGAGAATAAT (p.Ile227fs∗), was identified only in the proband and father, indicating that the mutation cosegregated with HOS phenotypes. The variant was validated by Sanger sequencing (Figure 2B, C). The novel mutation was predicted to cause a frameshift and the introduction of a stop codon (X) immediately after residue Ile 227 (p.I227fsX), resulting in a truncated protein of TBX5. As the carboxy terminal TAD domain was deleted in the truncated protein, we speculated that it no longer effectively activated the transcription of downstream targets. Thus, the mutation of *TBX5* (c.680_681insCTGAGAATAAT) identified in the family is a novel HOS–causing mutation.

## 4 Discussion

In the present study, we report information about a family of Chinese pedigree with HOS. The proband had severe upper limb deformities, which were bilateral and asymmetric, with the left being more severe than the right, as has been mentioned in many related studies (Basson et al., 1994; Heinritz et al., 2005; Terrett et al., 1994). Moreover, the proband displayed cardiac malformation: common atrium combined with unroofed coronary sinus syndrome and persistent left superior vena cava. To the best of our knowledge, such a cardiac malformation has rarely been reported in patients with HOS.

Through whole exome sequencing and genetic analysis, we identified a small–insertion mutation of *TBX5*, c.680_681insCTGAGAATAAT (p.I227fs∗). The human *TBX5* gene encodes T-box transcription factor 5 (TBX5) of 518 amino acids. The T-box, a highly evolutionarily conserved DNA binding motif, is located between amino acid residues 56 and 236. In addition, the TBX5 protein contains two nuclear localization signals (NLS1, aa 78–90; NLS2, aa 325–340), a nuclear export signal (NES, aa 152–160), and a C-terminal transactivation domain (TAD, aa 340–379) (Collavoli et al., 2003; Zaragoza et al., 2004). The novel mutation identified in our case was predicted to result in a truncated protein of TBX5 in which the TAD domain was deleted. Several nonsense mutations (p.Gln251∗, p.Arg279∗, p.Ser284∗, p.Tyr291∗, p.Glu316∗ and p.Gln362∗) in the region between the T-box domain and TAD domain have been reported to cause HOS. Since all these mutations lead to the deletion of the TAD domain, but retain the T-box domain, we infer that the TAD domain is also necessary to prevent the occurrence of HOS. In support of this notion, three missense mutations (p.Arg355Cys, p.Ser372Leu, and p.Gln376Arg) associated with HOS were found in the TAD domain. As TAD domain is essential for TBX5 to function as a transcriptional activator to regulate the expression of downstream targets in the development of the heart and forelimbs, we speculated that the truncated TBX5 resulting from the novel mutation has defects in activating the transcription of downstream targets (Agarwal et al., 2003; Bruneau et al., 2001).

The relationship between genotype and phenotype in individuals with HOS has always been a controversial issue. The proband described in this report inherited the mutation from his father. However, the HOS phenotypes of the proband were much more severe than those of his father. Consistent with our findings, some studies reported that HOS phenotypes display increasing severity in succeeding generations if the *TBX5* mutation is inherited in an autosomal manner (Fan et al., 2003; Newbury-Ecob et al., 1996). The mechanisms underlying this phenomenon remain unclear. It is possible that in the genotype–phenotype analysis for HOS, epigenetic analysis should be considered in the future.

In conclusion, this study described a new HOS case with rare congenital heart defects and severe bilateral upper limb deformities and identified a novel mutation of *TBX5*, c.680_681insCTGAGAATAAT (p.I227fs∗) as the pathogenic genetic lesion. Our findings expand the genetic aetiology spectrum of HOS and may be helpful for clinical genetic counselling of the family.

## Declarations

### Author contribution statement

All authors listed have significantly contributed to the investigation, development and writing of this article.

### Funding statement

This work was supported by the National Natural Science Foundation of China (81972038).

### Data availability statement

Data will be made available on request.

### Declaration of interest’s statement

The authors declare no conflict of interest.

### Additional information

No additional information is available for this paper.
